# Cytomegalovirus infection is a risk factor for venous thromboembolism in ANCA-associated vasculitis

**DOI:** 10.1186/s13075-022-02879-7

**Published:** 2022-08-10

**Authors:** C. King, R. Patel, C. Mendoza, J. K. Walker, E. Y. Wu, P. Moss, M. D. Morgan, D. O’Dell Bunch, L. Harper, D. Chanouzas

**Affiliations:** 1 Department of Renal Medicine, Queen Elizabeth Hospital, University Hospitals Birmingham, Birmingham, UK; 2 Institute of Immunology and Immunotherapy, University of Birmingham, Cancer Sciences Building, Edgbaston, Birmingham, B15 2TT UK; 3 University of North Carolina Gillings School of Global Public Health, Chapel Hill, NC USA; 4 University of North Carolina Pediatric Allergy, Immunology, and Rheumatology, Chapel Hill, USA; 5 Hull York Medical School, University of Hull, Hull, UK; 6 University of North Carolina Department of Medicine, Kidney Centre, Chapel Hill, NC USA; 7 Institute of Applied Health Research, University of Birmingham, Birmingham, UK; 8 Institute of Inflammation and Ageing, University of Birmingham, Birmingham, UK

**Keywords:** ANCA, Vasculitis, Cytomegalovirus, Thrombosis

## Abstract

**Background:**

Venous thromboembolism (VTE) is a common complication in patients with anti-neutrophil cytoplasm antibody (ANCA)-associated vasculitides (AAV) and confers significant morbidity and mortality. Both acute and past cytomegalovirus (CMV) infection have been identified as risk factors for VTE in immunocompetent and immunosuppressed individuals. Here, we examine whether past exposure to CMV is a risk factor for VTE amongst patients with AAV.

**Methods:**

We retrospectively analysed outcomes of patients with a new diagnosis of AAV from a UK cohort. All confirmed cases of VTE where CMV IgG serology was available were recorded. Retrospective collection of the same data for patients at a North American centre was used as a validation cohort.

**Results:**

VTE was common with 12% of patients from the study cohort (total 259 patients) developing an event during the median follow-up period of 8.5 years of which 60% occurred within the first 12 months following diagnosis. Sixteen percent of CMV seropositive patients developed a VTE compared with 5% of patients who were seronegative (*p* = 0.007) and CMV seropositivity remained an independent predictor of VTE in multivariable analysis (HR 2.96 [1.094–8.011] *p* = 0.033). CMV seropositivity at diagnosis was confirmed as a significant risk factor for VTE in the American validation cohort (*p* = 0.032).

**Conclusions:**

VTE is common in patients with AAV, especially within the first year of diagnosis. Past infection with CMV is an independent risk factor associated with VTE in AAV.

**Supplementary Information:**

The online version contains supplementary material available at 10.1186/s13075-022-02879-7.

## Background

The anti-neutrophil cytoplasm antibody (ANCA)-associated vasculitides (AAV) are a group of primary vasculitides localised to small and medium sized blood vessels and comprise granulomatosis with polyangiitis (GPA), microscopic polyangiitis (MPA) and eosinophilic granulomatosis with polyangiitis (EGPA) [1]. Although advances in the treatment of AAV have significantly improved survival, disease and treatment-related complications are common [2].

Venous thromboembolism (VTE) in patients with AAV is common with previous studies identifying a 4-fold increased risk in AAV compared with the general population. Furthermore, VTE adds to the considerable morbidity and mortality within this vulnerable population [3–8]. A number of studies have indicated that the highest risk of VTE occurs during periods of disease activity when inflammation is high, although patients with AAV remain hypercoagulable even during remission [3, 5–7, 9, 10]. Several mechanisms have been described to explain this thrombotic tendency associated with AAV. Neutrophil extracellular traps (NETs) and neutrophil-derived microparticles express tissue factor after stimulation by ANCA, causing activation of coagulation [11]. Anti-plasminogen antibodies in AAV patients impair fibrinolysis which may also contribute to thrombosis [12]. A recent prospective study of patients with AAV, who were enrolled during active disease and followed longitudinally, demonstrated that elevated microparticle tissue factor activity, and increased levels of anti-plasminogen antibodies in remission, were strong indicators of VTE [10]. Moreover, hypoalbuminaemia was also found to be significantly associated with VTE risk amongst patients with AAV.

Cytomegalovirus (CMV) is a herpes virus with a high prevalence that increases with age and socioeconomic deprivation. CMV results in an asymptomatic infection in most individuals but establishes a state of persistent infection. CMV is present in over half the population by middle age and is thought to undergo a state of latency with intermittent periods of viral reactivation [13]. CMV targets vessel wall monocytes, macrophages, smooth muscle cells and endothelial cells leading to a multi-step process of endothelial dysfunction [14]. The virus itself is also prothrombotic; it can initiate the generation of thrombin by having essential phospholipid (pro-PL) and tissue factor (TF) activities on its surface [15].

Interestingly, acute CMV infection has been identified as a risk factor for VTE in small studies both in immunocompetent and immunosuppressed individuals [9, 16–20]. A case control study in immunocompetent individuals showed that past CMV infection, as well as high titres of anti-CMV IgG and anti-CMV IgM antibodies, suggestive of the presence of viral reactivation, were all associated with the occurrence of VTE [18]. A small case series described 2 patients with AAV who developed 3 episodes of VTE in the context of CMV reactivation [21]. We have recently shown that intermittent CMV reactivation, without overt clinical disease, is common in patients with AAV, occurring in 25% of patients with AAV in stable remission over 1 year [22]. We anticipate that CMV reactivation during the acute phase of AAV, when patients are exposed to an intense period of inflammation and immunosuppression, will be even more frequent. However, it is not yet known whether chronic CMV infection predisposes to the development in VTE in this high-risk patient group.

In this retrospective analysis, we have examined whether past exposure to CMV, as evidenced by the presence of anti-CMV IgG antibodies, is a risk factor for the occurrence of VTE amongst patients with AAV.

## Methods

### Study design, setting and participants

We retrospectively analysed outcomes of patients with a diagnosis of AAV who attended the vasculitis clinics at University Hospitals Birmingham NHS Foundation Trust (UHBFT), Birmingham, UK from January 2014 to December 2015. Patients were included if they were serotyped for CMV IgG at diagnosis or during clinical follow-up. CMV IgG was assayed at diagnosis for those patients presenting with AAV between 2008 and 2013. All patients with a diagnosis of AAV prior to 2008 had their CMV IgG assayed in 2008 as per standard of care at the time.

We retrospectively analysed outcomes of consecutive patients with a new diagnosis of AAV, presenting between 2014 and 2019, at the University of North Carolina (UNC) Kidney Centre, Chapel Hill, North Carolina, USA, as a validation cohort. The patients from UNC all had CMV IgG assayed retrospectively from samples stored at time of diagnosis.

All patients met the European Medicines Agency vasculitis classification algorithm for AAV.

### Data collection

Data was collected retrospectively from electronic and paper medical records from UHBFT and UNC Kidney Centre. UHBFT data collection included demographics [age, gender, ethnicity, disease phenotype, treatment modality and organ involvement], laboratory parameters [biochemical: serum creatinine, estimated glomerular filtration rate (eGFR) calculated using MDRD, serum albumin, haemoglobin, C-reactive protein (CRP measured using the SpaPLus Assay, Binding Site, UK), and ANCA serotype] as well as dialysis requirement. Classical risk factors were recorded, including malignancy and the use of anticoagulation. We recorded all confirmed cases of VTE following the diagnosis of AAV. UNC Kidney Centre data collection included patient demographics and more limited laboratory parameters as well as VTE episodes since diagnosis.

### CMV serostatus determination

All patients were serotyped for CMV IgG using an Anti-CMV IgG enzyme-linked immunosorbent assay (ELISA). The titres of CMV IgG were not compared as this is not a quantitative assay.

### Statistics and analysis

The incidence of VTE associated with AAV was calculated as the number of VTEs occurring during 100 person-years of follow-up. For this calculation, we counted all VTEs in patients after diagnosis of AAV. We compared demographic, disease characteristics and risk factors in patients who developed a VTE associated with AAV and those who did not develop a VTE. Data are presented as medians and interquartile range (IQR). Differences in proportions between groups were tested using the Fisher’s exact test or chi-square test, when appropriate. Numerical data between groups were compared using the Mann–Whitney *U*-test or the *t*-test where appropriate. A two-sided *P*-value < 0.05 was considered to indicate statistical significance.

Log rank test was used to compare Kaplan-Meier survival curves for time to first VTE. Cox regression was used to assess the relationship between different characteristics and time to first VTE by univariable analysis. Those factors with a significance *p*-value < 0.05 were included in a multivariable Cox regression analysis.

## Results

### VTE in ANCA-associated vasculitis is common and more likely to occur at disease onset

Two hundred fifty-nine patients with AAV from our UK cohort (UHBFT) were included in the study and were followed for a median of 8.5 years (IQR 4.6–12.4 years). Patient demographics, disease characteristics and immunosuppressive induction treatment regimens are outlined in Table 1. All patients were treated with steroids and 78% received cyclophosphamide. Data on plasma exchange was not available.

**Table 1:** UHBFT patient demographics in relation to CMV serostatus

Characteristic	All patients ***n*** = 259	CMV status		
		CMV+ve patients ***n*** = 157	CMV−ve patients ***n*** = 102	***p*** value*
Median age at diagnosis in years (IQR)	58 (46–68)	62 (50–70)	50 (36–64)	< 0.001
Male gender	139 (54%)	85 (55%)	53 (52%)	0.657
GPA	160 (62%)	91 (58%)	69 (68%)	0.279
MPA	94 (36%)	63 (40%)	31 (30%)	
CSS	5 (2%)	3 (2%)	2 (2%)	
PR3 +ve	151 (58%)	90 (57%)	61 (60%)	0.693
MPO +ve	108 (42%)	67 (43%)	41 (40%)	
*Ethnicity:*				*0.304*
White British	229 (88%)	134 (85%)	95 (93%)	
Asian or Asian British-Pakistani	10 (4%)	7 (5%)	3 (3%)	
Asian or Asian British-Indian	7 (3%)	5 (3%)	2 (2%)	
Black or Black British-Caribbean	4 (2%)	4 (3%)	0	
Any other	9 (3%)	7 (4%)	2 (2%)	
*Induction treatment:*				
Cyclophosphamide	199 (78%)	119 (77%)	80 (79%)	0.718
Rituximab	8 (3%)	3 (2%)	5 (5%)	0.174
Cyclophosphamide and rituximab	4 (2%)	3 (2%)	1 (1%)	0.553
Azathioprine	18 (7%)	10 (6%)	8 (8%)	0.648
Mycophenolate mofetil	15 (6%)	15 (10%)	0	0.001
Methotrexate	7 (3%)	4 (3%)	3 (3%)	0.848
*Organ involvement:*				
Renal	179 (69%)	115 (73%)	64 (63%)	0.074
ENT	62 (24%)	33 (21%)	29 (28%)	0.172
Pulmonary haemorrhage	24 (9%)	17 (11%)	7 (7%)	0.282
Lung	68 (26%)	42 (27%)	26 (25%)	0.822
Nervous system	22 (8%)	16 (10%)	6 (6%)	0.224
Eye	22 (8%)	13 (8%)	9 (9%)	0.878
Median creatinine at diagnosis μmol/L (IQR)	173 (92–407)	212 (100–422)	142 (87–374)	0.107
Dialysis requirement at diagnosis or during follow-up	45 (17%)	32 (20%)	13 (13%)	0.113
Median duration of follow-up in years (IQR)	8.5 (4.6–12.4)	8.3 (4.2–12.2)	8.9 (5.5–12.7)	0.136
Median CRP at diagnosis mg/L (IQR)	58 (16–139)	65 (20–152)	42 (12–112)	0.052
Median urine ACR at diagnosis mg/mmol (IQR)	36 (2–132)	47 (3–185)	23 (1–93)	0.069
Median serum albumin at diagnosis g/L (IQR)	35 (30–40)	35 (29–39)	36 (30–42)	0.085

As in other studies, VTE was common with 12% of patients developing an event during the follow-up period; the incidence of VTE was 1.4/100 patient-years. Thirty-six episodes of VTE occurred with 17 deep vein thromboses and 19 pulmonary emboli. Median time to first VTE event was 147 days (IQR 45–1700 days). There was only one episode of DVT provoked by a dialysis catheter. As previously reported, the highest risk for a VTE was around the time of disease activity with 12 events occurring in the first 90 days following diagnosis and a total of 22 events in the first year. The incidence increased to 6.9/100 person-years during active disease within the 12 months of diagnosis.

There was no difference in patient demographics or disease characteristics including treatment modality, between those patients who had a VTE and those that did not develop a VTE, as outlined in Table 2. There was no difference in known risk factors for VTE including previous VTE, malignancy or warfarin use at diagnosis in those who had a VTE compared to those without.

**Table 2:** Comparison between UHBFT patients that experienced a VTE episode during follow-up versus those that did not

Characteristic	VTE status		
	Patients with no VTE ***n*** = 229	Patients with VTE ***n*** = 30	***p*** value
Median age at diagnosis in years (IQR)	57 (45–68)	61 (52–69)	0.235
Male gender	120 (52%)	19 (63%)	0.259
GPA	142 (62%)	18 (60%)	0.670
MPA	82 (36%)	12 (40%)	
CSS	5 (2%)	0	
PR3 +ve	131 (57%)	20 (67%)	0.323
MPO +ve	98 (43%)	10 (33%)	
Caucasian ethnicity	207 (90%)	27 (90%)	0.945
CMV seropositive	132 (58%)	25 (83%)	0.007
*Induction treatment:*			
Cyclophosphamide	176 (78%)	23 (85%)	0.343
Rituximab	8 (4%)	0	0.322
Cyclophosphamide and rituximab	4 (2%)	0	0.487
Azathioprine	17 (7%)	1 (4%)	0.469
Mycophenolate mofetil	12 (5%)	3 (11%)	0.225
Methotrexate	7 (3%)	0	0.355
*Organ involvement*			
Renal	154 (67%)	25 (83%)	0.073
ENT	58 (25%)	4 (13%)	0.148
Pulmonary haemorrhage	23 (10%)	1 (3%)	0.233
Lung	64 (28%)	4 (13%)	0.087
Nervous system	20 (9%)	2 (7%)	0.703
Eye	21 (9%)	1 (3%)	0.281
Dialysis requirement at diagnosis or during follow-up	35 (13%)	10 (33%)	0.014
Median creatinine at diagnosis μmol/L (IQR)	164 (91–389)	241 (163–593)	0.062
Median CRP at diagnosis mg/L (IQR)	44 (14–132)	139 (63–244)	< 0.001
Median urine ACR at diagnosis mg/mmol (IQR)	31 (2–120)	90 (16–314)	0.112
Median serum albumin at diagnosis g/L (IQR)	35 (30–40)	33 (29–37)	0.107
Median Hb at diagnosis g/L (IQR)	100 (88–122)	96 (88–118)	0.313
VTE event pre-AAV diagnosis	5 (2%)	2 (7%)	0.154
Malignancy at diagnosis of AAV	3 (1%)	1 (3%)	0.398
Malignancy at diagnosis of VTE or end of f/u	24 (11%)	2 (7%)	0.513
Warfarin at diagnosis of AAV	3 (1%)	1 (3%)	0.392

### VTE is associated with high CRP and dialysis dependence

Patients with AAV who developed a VTE had higher levels of inflammation at diagnosis with a median CRP of 139 mg/L (IQR 63–244) vs. 44 mg/L (14–132) in those without VTE (*p* < 0.001) (Table 2). Patients with AAV who developed a VTE were significantly more likely to be dialysis dependent at presentation or during follow-up than those who did not (33% vs 13%; *p* = 0.014). Patients that developed VTE during follow-up also had higher serum creatinine at diagnosis compared to those that did not develop VTE, although this difference was not significant [median serum creatinine 241 μmol/L (IQR 1.63–593) vs 164 μmol/L (91–389); (*p* = 0.062)].

### VTE is strongly associated with CMV seropositivity

**Fig. 1:**
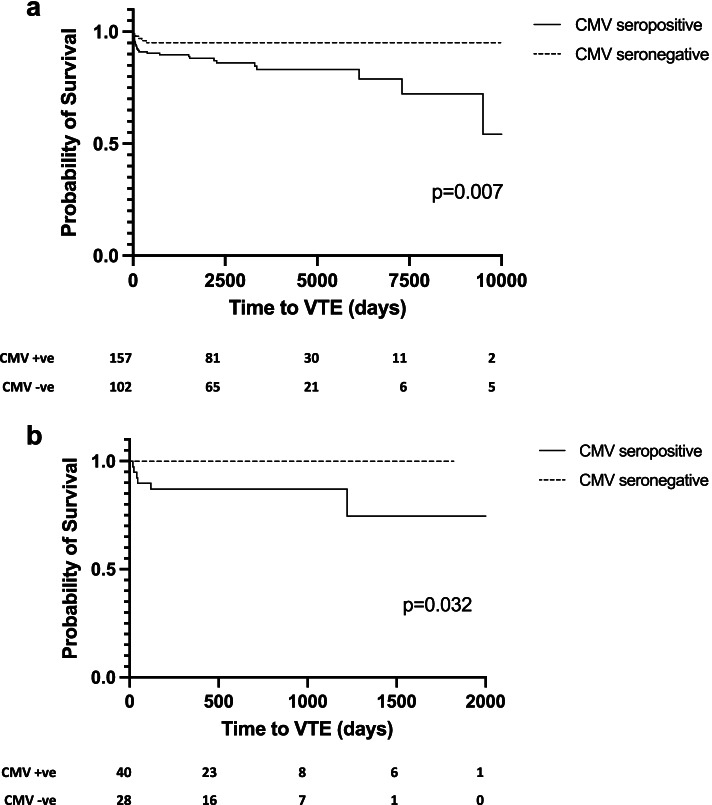
**a** Time to VTE episode in CMV-seropositive versus CMV-seronegative UHBFT AAV patients. **b** Time to VTE episode in CMV-seropositive versus CMV-seronegative UNC AAV patients. Time to VTE event was examined by Kaplan-Meier curve analysis (log rank test). CMV seropositive patients are shown in the solid line and CMV seronegative patients in the dashed line. Numbers of patients at risk for each time point displayed below the curve

CMV IgG was positive at diagnosis in 157 of 259 patients (61%). The duration of follow-up in those who were CMV seropositive (median 8.3 years) and those who were CMV seronegative (median 8.7 years) was not significantly different (*p* = 0.136). There was an increased risk of VTE in those who were CMV IgG seropositive; 25 (16%) CMV seropositive patients had a VTE episode compared with 5 (5%) CMV seronegative patients (*p* = 0.007). All episodes of VTE in CMV seronegative patients occurred during the first 12 months from disease diagnosis (Fig. 1a). In CMV seropositive patients, just over half of VTE events (16 episodes) occurred during the first 12 months. CMV seropositive patients continued to develop VTE during the follow-up period (14 episodes).

Univariable Cox regression analysis in our UHBFT cohort identified CRP at diagnosis, CMV seropositivity, dialysis requirement, age and the absence of ear nose and throat (ENT) organ involvement as significant risk factors for VTE (*p* = < 0.05) after diagnosis of AAV (Table 3). Univariable analysis results for all other variables are listed in the supplemental material table 1.

**Table 3:** Factors associated with VTE by univariable and multivariable analysis in UHBFT patients

Variable	Univariable analysis*		Multivariable analysis	
	HR (confidence interval)	***p*** value	HR (confidence interval)	***p*** value
Age at diagnosis in years	1.026 (1.005–1.049)	0.016	1.009 (0.984–1.034)	0.479
CMV seropositive	3.485 (1.449–8.386)	0.005	2.960 (1.094–8.011)	0.033
Dialysis requirement at diagnosis or during follow-up	2.586 (1.267–5.276)	0.009	2.081 (0.962–4.499)	0.063
CRP at diagnosis mg/L	1.005 (1.003–1.008)	< 0.001	1.005 (1.002–1.008)	0.001
ENT organ involvement	0.377 (0.118–0.960)	0.042	0.686 (0.201–2.334)	0.546

Cox regression analysis used

*Only those variables with a *p* value < 0.05 after univariable analysis are included in this table. All other variables are included in the supplemental material

In a multivariable Cox regression analysis, CMV seropositivity (HR 2.960 [1.094–8.011] *p* = 0.033) and the level of inflammation at diagnosis (CRP at diagnosis per 1mg/l HR 1.005 [1.002–1.008] *p* = 0.001) were independent predictors of VTE (Table 3). Thus, CMV seropositivity remained a strong determinant of VTE after controlling for other variables.

### Confirmation of strong association between CMV seropositivity and VTE in validation cohort

The validation cohort comprised of 68 patients from a North American patient cohort (UNC Kidney Centre) with a median follow-up of 1.6 years (IQR 1.1–2.6). Patient demographics and disease characteristics were similar to the UHBFT cohort including organ involvement, creatinine and dialysis dependency at diagnosis. However, more patients were MPO positive (60%) compared to the UHBFT cohort (42%) and induction treatment included either cyclophosphamide (12%), rituximab (38%) or both of these in combination (46%), alongside steroids. Patient demographics and disease characteristics are outlined in the supplemental material table 2. VTE occurred in 9% of patients with a median time to first VTE episode of 43 days (IQR 23–395); the incidence of VTE was 4.7/100 person-years. In this smaller cohort with a shorter duration of follow-up, only 6 VTE episodes occurred. The data from the validation cohort confirmed that CMV seropositivity at diagnosis was a significant risk factor for VTE as displayed in the Kaplan-Meier survival curve (Fig. 1b). Fifteen percent of CMV IgG seropositive UNC Kidney Centre patients had a VTE whereas VTE was not seen amongst CMV seronegative patients (*p* = 0.032). UNC Kidney Centre patients who developed a VTE were also more likely to be dialysis dependent and have a higher creatinine at diagnosis although these differences were not significant in this smaller cohort (supplemental material table 3).

### VTE after 12 months is associated with relapse or increased inflammation

Whilst the majority of VTE episodes occurred within the first 12 months following diagnosis of AAV when inflammation is high, 14 out of a total of 36 episodes from our UHBFT cohort occurred after 12 months (39% episodes) (Table 4). The median time to VTE episode, for those occurring > 12 months after AAV diagnosis, was 6.5 years (IQR 3.4–13.0). Importantly, VTE episodes after the first 12 months were only seen amongst CMV seropositive patients. Out of these 14 episodes, 36% occurred during a clinical vasculitis relapse, 29% occurred during an episode of infection requiring hospitalisation and 21% were associated with malignancy. The median CRP at the time of VTE episode was 42mg/L (IQR 27–122) compared to a median CRP at diagnosis of AAV of 58mg/L (16–139), suggesting that inflammation at the time of these late VTE episodes was also high.

**Table 4:** Patients with a VTE episode > 12 months after initial diagnosis of AAV

Characteristic	All episodes ***n*** = 14
CMV seropositive	14 (100%)
Clinical relapse at time of VTE	5 (36%)
Infection requiring hospitalisation at time of VTE	4 (29%)
Malignancy at time of VTE	3 (21%)
Median creatinine at time of VTE in μmol/L (IQR)	143 (110–188)
Median CRP at time of VTE in mg/L	42 (27–122)
Median urine ACR at time of VTE mg/mmol (IQR)	5 (1–8)
Median serum albumin at time of VTE g/L (IQR)	38 (33–46)
Median serum Hb at time of VTE g/L (IQR)	119 (93–126)
Median time to VTE event in years (IQR)	6.5 (3.4–13.0)

## Discussion

Our study shows that the incidence of VTE in patients with AAV is high at 12% and 1.4/100 person-year, consistent with an increasing body of evidence from previous studies in AAV where VTE incidence ranged from 9 to 18% [4–7]. Our study is the first to identify that CMV seropositivity is an independent risk factor for VTE in AAV with a HR of 2.960 (1.094–8.011) on multivariable analysis. Furthermore, we confirmed the association between CMV seropositivity and VTE in an independent validation cohort of AAV.

The incidence of VTE in our study was higher than previously shown in the general population with an age range corresponding to that of AAV patients (0.31 cases/100 person-years) [23]. Sixty-one percent of the VTE episodes occurred within the first 12 months following diagnosis of AAV where disease activity and inflammation are high. This temporal relationship has also been observed in other recent studies suggesting that inflammation is associated with a pro-thrombotic state [3, 4]. The majority of the VTE episodes that occurred later in the disease course were around the time of severe infection, malignancy or relapse of AAV, all of which are associated with heightened inflammation that may promote thrombosis.

Recent studies have addressed risk factors for VTE in AAV, but none have explored CMV seropositivity as a risk factor. To date, this has only been described in a small case series involving 2 patients with AAV with three episodes of thrombosis following CMV reactivation [21].

A number of mechanisms for VTE associated with CMV reactivation have been proposed. Direct infection of vascular endothelial cells increases the release of von Willebrand factor and expression of TF. The virus itself is prothrombotic generating pro-PL and TF on its surface and the formation of anti-phospholipid antibodies in response to CMV infection [15, 17]. With regard to VTE and AAV, given that the endothelial cells play a central role in coagulation, injury to vessels from leucocytes activated by ANCA as well as reactivated CMV may further exacerbate a thrombotic tendency [21].

In a previous proof-of-concept study, we have shown that asymptomatic CMV reactivation is common amongst AAV patients in remission, occurring in 1 in 4 CMV seropositive patients over a 12-month period [22]. Moreover, anti-viral treatment suppresses asymptomatic CMV reactivation in these patients. We have also demonstrated that asymptomatic reactivation of CMV is associated with the expansion of an endothelial homing cytotoxic T cell subset known as CD4^+^CD28^null^ T cells, exclusively seen in CMV seropositive patients. It is possible that endothelial damage driven by CD4^+^CD28^null^ T cells is one of the factors leading to the pro-coagulant tendency associated with CMV infection. Our previous studies suggest that the CD4^+^CD28^null^ T cells percentage within peripheral blood is a surrogate marker for the intensity and frequency of subclinical asymptomatic CMV reactivation [14, 22]. We have previously observed that the proportion of CD4^+^CD28^null^ T cells increases during the first year in patients with AAV. However, the degree to which subclinical reactivation of CMV occurs during the acute phase of AAV is not currently known [24]. We anticipate that viral reactivation will be significantly higher during the acute phase of the disease process, at a time where patients are exposed to intensive immunosuppressive therapy and heightened inflammation [25, 26]. A limitation of our current study described here is that we were not able to measure CMV viral titres in CMV seropositive individuals. Our present study shows that CMV seropositive patients were almost 3 times more likely to develop a VTE episode compared to CMV seronegative patients on multivariable analysis. We found that VTE episodes after 12 months following a new diagnosis of AAV were associated with inflammation in that they appeared to occur in association with a disease relapse, severe infection or elevated CRP. VTE episodes after 12 months were only seen in CMV seropositive patients. Whilst we cannot prove causality, we hypothesise that during periods of heightened inflammation, CMV reactivation in CMV seropositive individuals may lead to further endothelial damage and pro-coagulant tendency through mechanisms such as expansion of endothelial homing CD4^+^CD28^null^ T cells thereby contributing to the development of VTE. We are currently undertaking an observational study investigating the frequency and magnitude of asymptomatic CMV reactivation in patients with a new diagnosis or relapse of AAV and its impact on clinical outcomes (clinicaltrials.gov registration 294850). In that study, CMV viral load is being measured at regular intervals during active disease and correlated with clinical outcomes including episodes of VTE and plasma markers of pro-coagulant activity such as tissue factor expression.

In addition to CMV seropositivity, we identified CRP, age, dialysis dependency and lack of ENT involvement as significant factors associated with VTE on univariable analysis. However, the level of CRP at diagnosis was the only other risk factor, in addition to CMV seropositivity, that remained a significant predictor of VTE on multivariable analysis. Similarly, Kronbichler et al. also identified higher CRP and higher creatinine at baseline as independent predictors of VTE in a large study including 417 patients with AAV enrolled onto European Vasculitis Society (EUVAS) randomised control trials [4]. Other studies have highlighted a higher Birmingham vasculitis activity score (BVAS) or specific organ involvement (gastrointestinal, cardiac, pulmonary haemorrhage) as independent risk factors [4, 5, 20]. We were not able to collect data on BVAS scores in this retrospective study, but we were able to assess organ involvement at the time of diagnosis. ENT involvement was associated with a lower incidence of VTE on univariable analysis which may reflect reduced systemic inflammation in these patients.

Our study is retrospective which is a key limitation. AAV is a rare disease, so a prospective study with a large enough number of patients to detect significance in risk factors for VTE events would be challenging. We only included confirmed VTE events in our analyses, i.e. the AAV patient cohorts were not investigated in a protocolised, prospective manner for VTE. However, arguably, these are the events which are likely to be most clinically relevant. Approximately half of the UHBFT cohort had their CMV serostatus assayed following disease diagnosis. Given that AAV is primarily a disease of older adults, we anticipate that the CMV serostatus of those individuals is unlikely to have altered in the intervening period but cannot be certain of this.

The number of VTE events within the study was relatively low and adjustments for multiple comparisons in the multivariable analysis was therefore restricted. Amongst the UNC Kidney centre cohort, the number of VTE cases was even smaller due to a smaller cohort size and shorter available follow-up. Additionally, missing data values on some variables, such as serum albumin, proteinuria and CRP, limited further analysis of risk factors. Only 1 patient with a VTE from the UNC cohort had a CRP value so comparison for this was not possible in this cohort. We also did not have any data available on some medications which are known risk factors for VTE including hormone replacement therapy, oral contraceptive use or VTE prophylaxis treatment in hospitalised patients.

A strength of our study is the additional UNC Kidney Centre patient cohort validating CMV seropositivity as a risk factor for thrombosis in AAV. This cohort was comparable to the UHBFT cohort with respect to patient demographics (age, gender) and disease presentation (creatinine, CRP, albumin at diagnosis and dialysis dependency). Due to a low number of VTE episodes, we were unable to conduct multivariable analysis on the validation cohort.

## Conclusions

VTE is common in patients with AAV, especially within the first year of diagnosis, contributing to the morbidity and mortality associated with the disease. CMV seropositivity is an independent risk factor associated with increased development of VTE amongst patients with AAV.

## Supplementary Information

**Additional file 1: Supplemental Table 1.** Factors associated with VTE by univariable analysis in UHBFT patients. **Supplemental Table 2.** UNC Kidney Centre patient demographics in relation to CMV serostatus. **Supplemental Table 3.** Comparison between UNK Kidney Centre patients that experienced a VTA episode during follow up versus those that did not.

## Data Availability

The datasets used and/or analysed during the current study are available from the corresponding author on reasonable request.

## Abbreviations

AAV: Anti-neutrophil cytoplasm antibody (ANCA)-associated vasculitides
VTE: Venous thromboembolism
CMV: Cytomegalovirus
pro-PL: Essential phospholipid
TF: Tissue factor
UHBFT: University Hospitals Birmingham NHS Foundation Trust
UNC: University of North Carolina
eGFR: Estimated glomerular filtration rate
CRP: C-reactive protein
IQR: Interquartile range
BVAS: Birmingham vasculitis activity score
