# Supplementary material for: Cytomegalovirus infection is a risk factor for venous thromboembolism in ANCA-associated vasculitis
Source: Arthritis Res Ther. 2022 Aug 10;24:192. doi: 10.1186/s13075-022-02879-7 (PMC9364516; doi:10.1186/s13075-022-02879-7)
Supplement: Supplementary file 1 — Additional file 1: Supplemental Table 1. Factors associated with VTE by univariable analysis in UHBFT patients. Supplemental Table 2. UNC Kidney Centre patient demographics in relation to CMV serostatus. Supplemental Table 3. Comparison between UNK Kidney Centre patients that experienced a VTA episode during follow up versus those that did not. [file 13075_2022_2879_MOESM1_ESM.pdf]

Supplemental material

Supplemental table 1: Factors associated with VTE by univariable analysis in UHBFT patients

Supplemental table 2: UNC Kidney Centre patient demographics in relation to CMV serostatus

Supplemental table 3: Comparison between UNK Kidney Centre patients that experienced a VTE episode during follow up versus those that did not

Supplemental table 1: Factors associated with VTE by univariable analysis in UHBFT patients:

Variable	HR (Confidence interval)	P value
PR3 versus MPO disease	1.226 (0.610-2.463)	0.567
Induction treatment immunosuppressive agent	0.986 (0.739-1.315)	0.924
Organ involvement:		
Renal	2.142 (0.936-4.901)	0.071
Pulmonary haemorrhage	0.315 (0.043-2.300)	0.255
Lung	0.488 (0.139-1.258)	0.138
Nervous system	0.963 (0.295-3.147)	0.951
Eye	0.303 (0.041-2.210)	0.239
Creatinine at diagnosis umol/L (IQR)	1.001 (1.000-1.001)	0.135
Urine ACR at diagnosis mg/mmol (IQR)	1.001 (0.999-1.002)	0.299
Serum albumin at diagnosis g/L (IQR)	0.951 (0.902-1.003)	0.067
Hb at diagnosis g/L (IQR)	0.992 (0.980-1.004)	0.183
VTE event pre-AAV diagnosis	2.476 (0.593-10.349)	0.214
Malignancy at diagnosis of AAV	1.370 (0.184-10.182)	0.759
Malignancy at diagnosis of VTE or end of f/u	0.422 (0.101-1.772)	0.239
Warfarin at diagnosis of AAV	2.422 (0.329-17.827)	0.385

Supplemental table 2: UNC Kidney Centre patient demographics in relation to CMV serostatus:

Characteristic	All Patients n=68 (IQR)	CMV status		
		CMV+ve patients n= 40	CMV-ve patients n= 28	P value
Median age at diagnosis (years)	61 (47-71)	66 (52-74)	51 (37-65)	<0.001
Male gender	40 (59%)	24 (60%)	16 (57%)	0.814
PR3 +ve	27 (40%)	13 (33%)	14 (50%)	0.147
MPO +ve	41 (60%)	27 (67%)	14 (50%)	
Ethnicity:				
White	61 (90%)	135 (87%)	26 (93%)	0.483
Black	5 (7%)	3 (8%)	2 (7%)	
Hispanic	2 (3%)	2 (5%)	0 (0%)	
Induction treatment:				
Cyclophosphamide	8 (12%)	5 (13%)	3 (11%)	0.822
Rituximab	26 (38%)	19 (48%)	7 (25%)	0.060
Cyclophosphamide and Rituximab	31 (46%)	15 (38%)	16 (57%)	0.109
Plasma exchange	25 (37%)	14 (35%)	11 (39%)	0.718
Organ involvement:				
Renal	50 (74%)	31 (78%)	19 (68%)	0.375
ENT	30 (44%)	17 (43%)	13 (46%)	0.748
Lung	27 (40%)	17 (43%)	10 (36%)	0.574
Nervous system	7 (10%)	2 (5%)	5 (18%)	0.086
Median creatinine at diagnosis (umol/L)	184 (84-389)	210 (84-466)	165 (84-293)	0.386
Dialysis requirement at diagnosis or during follow up	15 (22%)	12 (30%)	3 (11%)	0.059
Median duration of follow up (years)	1.6 (1.1-2.6)	1.6 (1.2-2.3)	1.7 (1.1-2.8)	0.994
Median CRP at diagnosis mg/L (IQR)*	31 (4-126)	31 (5-138)	44 (2-126)	0.618
Median serum albumin at diagnosis g/L (IQR)	33 (28-38)	35 (29-40)	30 (27-35)	0.094

Supplemental table 3: Comparison between UNC Kidney Centre patients that experienced a VTE episode during follow up versus those that did not:

**Characteristic**	**VTE status**		
	**Patients with no VTE n=62**	**Patients with VTE n=6**	**P value**
Median age at diagnosis in years (IQR)	61 (46-69)	67 (46-82)	0.331
Male gender	35 (57%)	5 (83%)	0.201
PR3 +ve	24 (39%)	3 (50%)	0.589
MPO +ve	38 (61%)	3 (50%)	
Caucasian ethnicity	55 (89%)	6 (100%)	0.385
CMV seropositive	34 (55%)	6 (100%)	0.032
Induction treatment:			
Cyclophosphamide	8 (13%)	0	0.349
Rituximab	23 (38%)	2 (33%)	0.796
Cyclophosphamide and Rituximab	27 (44%)	4 (67%)	0.278
Plasma exchange	21 (34%)	4 (67%)	0.112
Organ involvement			
Renal	44 (71%)	6 (100%)	0.124
ENT	28 (45%)	2 (33%)	0.577
Lung	24 (39%)	3 (50%)	0.589
Nervous system	7 (11%)	0	0.385
Dialysis requirement at diagnosis or during follow up	12 (19%)	3 (50%)	0.139
Median creatinine at diagnosis umol/L (IQR)	175 (82-375)	442 (144-531)	0.198
Median serum albumin at diagnosis g/L (IQR)	33 (28-38)	31 (25-41)	0.664
Median Hb at diagnosis in g/L (IQR)	106 (88-124)	94 (86-108)	0.328
